# Internet of Things-Based ECG and Vitals Healthcare Monitoring System

**DOI:** 10.3390/mi13122153

**Published:** 2022-12-06

**Authors:** James Heaney, Jamie Buick, Muhammad Usman Hadi, Navneet Soin

**Affiliations:** School of Engineering, Ulster University, Newtownabbey BT37 0QB, Northern Ireland, UK

**Keywords:** ECG monitoring system, smart monitoring, heart diseases, cardiovascular diseases, IoT, sensors

## Abstract

Health monitoring and its associated technologies have gained enormous importance over the past few years. The electrocardiogram (ECG) has long been a popular tool for assessing and diagnosing cardiovascular diseases (CVDs). Since the literature on ECG monitoring devices is growing at an exponential rate, it is becoming difficult for researchers and healthcare professionals to select, compare, and assess the systems that meet their demands while also meeting the monitoring standards. This emphasizes the necessity for a reliable reference to guide the design, categorization, and analysis of ECG monitoring systems, which will benefit both academics and practitioners. We present a complete ECG monitoring system in this work, describing the design stages and implementation of an end-to-end solution for capturing and displaying the patient’s heart signals, heart rate, blood oxygen levels, and body temperature. The data will be presented on an OLED display, a developed Android application as well as in MATLAB via serial communication. The Internet of Things (IoT) approaches have a clear advantage in tackling the problem of heart disease patient care as they can transform the service mode into a widespread one and alert the healthcare services based on the patient’s physical condition. Keeping this in mind, there is also the addition of a web server for monitoring the patient’s status via WiFi. The prototype, which is compliant with the electrical safety regulations and medical equipment design, was further benchmarked against a commercially available off-the-shelf device, and showed an excellent accuracy of 99.56%.

## 1. Introduction

The use of Internet of Things (IoT) sensors for assessing the human body’s vital signs has grown in prominence in recent years [1]. While the primary driver for such devices has been ubiquitous on-demand healthcare monitoring, there is an increasing case for their use in enabling and improving the healthcare provisions in low- and middle-income countries (LMICs) [2,3]. The existing healthcare facilities in LMICs range from being inadequate to virtually non-existent, and this stems from a lack of finance, infrastructure, and trained manpower amongst other systemic factors [3]. As a result, a significant population and many communities are often deprived of medical care which invariably is expensive and inaccessible. At the same time, chronic disorders such as hypertension, diabetes, heart disease, and chronic obstructive pulmonary disease are becoming more prevalent [4]. In fact, cardiovascular diseases (CVDs) accounting for nearly 17.9 million deaths per year are now the leading cause of mortality across the world [4]. According to the World Health Organization (WHO), in 2016, CVDs accounted for more than 840,000 mortalities in the United States alone [4,5,6]. Similarly, according to the 2017 edition of the European Cardiovascular Disease Statistics, CVDs led to over 5.7 million deaths across Europe and European Union (EU), accounting for nearly 40% of all fatalities [7]. Globally, nearly 80% of CVD deaths occur in LMICs, where often the burden of disease is the greatest [8]. The numbers, as startling as they are, highlight the worldwide prevalence of CVDs, and they also bring to light how LMICs with the least resources suffer most of the disease burden.

While the continuous monitoring of CVD patients’ essential long-term physiological indicators (such as N-terminal pro B-type natriuretic peptide (NT-proBNP)) is crucial [9], day-to-day continuous heart rate monitoring and heartbeat/heartrate detection is equally important in the prognosis and understanding of the progress of the disease [2,3,10]. As such, the capture and analysis of the physiological electrocardiogram (ECG) signals have become the primary de facto healthcare monitoring tool for CVDs. Since the earliest bulky systems, the dramatic evolution of ECG monitors has been driven by advances in the system-on-chip (SoC) design and smart enabling technologies to now provide on-demand wearable-based ECG diagnostics [11,12,13]. As such, ECG monitoring devices are now employed in triaging in hospitals [14], care homes [15], outpatient ambulatory settings [16,17], and remote settings [18,19]. Underpinned by technologies, including the IoT [20,21], edge computing [22,23], and mobile computing [24,25], the ECG systems also incorporate a variety of computational parameters, such as processing frequencies, monitoring techniques, and third-person alerting systems [3,10,26]. Other than CVD detection and management, they have evolved to serve a variety of goals and objectives, including everyday activities [27], sports [28], and even mood-related reasons [29,30]. While decentralization of the diagnostics tools has been the norm in developed societies, the centralised in-patient diagnostics remain the least favoured option in LMICs, where the explicit (test costs) and implicit costs (travel time and loss of earnings) are often detrimental for the patients [3]. As such, the day-to-day monitoring via the physiological ECG signals can provide a comprehensive paradigm for assessing CVDs, aiding disease control and prevention via the continuous monitoring and analysis of the ECG data [2,3,10]. However, it is understood that often the medical infrastructure in LMICs makes the timely intervention and ongoing diagnosis of CVDs challenging owing to the lack of basic diagnostic modalities such as ECG. While the twelve-lead systems remain the gold standard for diagnosing the patient’s cardiac health and future treatment pathway, the commercially available single-lead ECG systems (two- or three-electrodes) have been tested in community settings wherein they have displayed high sensitivity (~98%) and sufficient specificity (~74%) to capture irregular heart rhythms [31]. For the LMICs, it can be argued that the lack of trained healthcare workers necessitates equipment which is end-user friendly, and thus, single-lead ECG systems are preferable to overcome the electrode placement and interpretation issues of multiple-lead systems [32,33]. While there are a number of commercial solutions available on the market (see Table 1) based on single-lead ECG acquisition as well as in the wearable domain, the end-user affordability is the underpinning driving factor for their uptake in LMICs. Consequently, there is a need for effective mitigation, low-cost, bespoke wireless portable health monitoring systems are required, which are reliable, accurate, and energy efficient.

Considering the widespread nature and significance of the problem, and to promote better understanding, analysis, design, and validation of the ECG monitoring systems from a larger viewpoint, we have developed a complete, end-to-end dual-mode (patient- and healthcare practitioner-centric) ECG monitoring device. The system utilises an AD8232 microchip (Analog Devices, USA) as the analog front-end which is then fed into an Arduino (MKR1010, Arduino, Italy) which offers both Bluetooth and WiFi connectivity to a smartphone or another external device for the remote monitoring of the patients. The system also comes equipped with temperature and specific oxygen (SpO_2_) sensors, and it can be powered by a rechargeable 3.7 V LiPo battery. The proposed device has the following specifications:

1. The device must display an ECG signal on a local display screen, and it records the data on a server.
2. To show the signal on a custom mobile application, the signal must be delivered over Bluetooth.
3. It should include capabilities that enable MATLAB integration; the ECG signal should be sent to MATLAB for further analysis. R-wave detection must be used to compute and display the heart rate on all of the display outputs.
4. All the user interfaces should record and show additional information such as blood oxygen level (SpO_2_) and skin temperature. The sensor data should be delivered via a WiFi connection to a ThingSpeak server.
5. The final prototype design should be printed on a high-quality PCB and housed in a protective casing. The device must be both portable and wireless.
6. The device must include a low-power sleep mode to enhance the battery’s lifetime and enhance the product’s portability requirements.

## 2. Materials and Methods

Here, we discuss the theoretical aspects which underpin the practical considerations employed in the system design.

### 2.1. Analog Front End (AFE)

**Figure 1:**
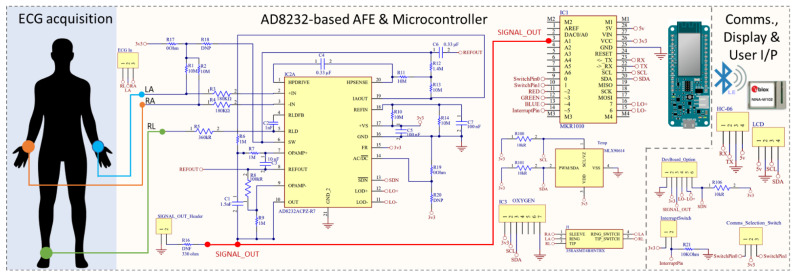
Custom-built, end-to-end ECG capturing system. The main components of the prototype are the AD8232-based AFE, temperature sensor (MLX90614), SpO2 sensor (MAX30100), and the Arduino MKR1010. The communication and display options are provided by the OLED and classic BT. The entire system is powered by a 3.7 V rechargeable Li battery.

The low amplitude raw ECG signal necessitates amplification [34], while the elimination of the noise components arising from a range of sources demands strong attenuation (see Figure 1). To eliminate any noise components that were recorded during the ECG capture, the amplified signal needs to pass through a filter. To reduce the AC noise produced by the mains power—50 Hz in Europe and 60 Hz in America—a band-pass or notch filter is generally utilised. If the patient is not motionless throughout the recording, an electromyogram-type noise is created, which alongside the respiration, might cause baseline drift. This is in addition to the electrode offset noise which can occur owing to the poor connection between the patient’s skin and the electrodes. To ensure that the ECG signal is suitable for the analysis, all these noise components must be removed or attenuated significantly prior to digitisation [10,26,35].

While there are several off-the-shelf fully integrated ECG front ends (see Table 2), based on the low-power consumption and low-cost criteria, we have utilised the AD8232 integrated signal conditioning block [10,36,37] (Analog Devices, USA). The AD8232 has a voltage range of 2–3.5 V and a low supply current of 170 µA, making it simple to integrate with an external microcontroller such as Arduino [38]. Moreover, the 4 mm × 4 mm packaging enables it to have a small PCB design, and it features a shutdown pin, allowing it to be configured to save the power. Additionally, the integrated circuit includes a two-pole adjustable high-pass/low-pass filtering arrangement as well as an adjustable gain (maximum gain of 100 V/V), allowing for the configurable capabilities to be tailored for performance testing. There are several features in the AD8232 including a right-leg driver (RLD), leads-off detection, and an in-built fast restore circuit. Given the safety considerations and requirements for a high signal-to-noise (SNR) ratio, a three-electrode design comprising of RA, LA, and RL (RLD) was chosen wherein the RLD/ground-electrode minimises the effects of common noise by extracting the common-mode voltage and driving an opposing signal into the patient. The 1 nF integrator capacitor (C2) works in conjunction with an internal 150 kΩ resistor for driving the RLD. To ensure the patient’s safety, a 360 kΩ resistor (R5) was used to ensure that the current was limited to less than 10 µA. For the three-electrode configuration, the DC leads-off detection mode works by setting the $\mathrm{AC}/\bar{\mathrm{DC}}$ line to ground via the R19 resistor (see Figure 1). The leads-off mechanism relies on pull-up resistors (R1, R2) connected to the positive supply (3V3), and it senses when either of the instrumentation amplifier input voltage is within 0.5 V from the positive rail. Thus, the leads-off detection pins remain low if the electrodes are connected correctly, and this will change to high when they are disconnected.

### 2.2. Choice of Microcontroller

A microcontroller is required to process the signal that has been amplified and filtered by the front end. To avoid the necessity for an external analogue-to-digital converter (ADC), such as the MCP3208, a microcontroller with an on-board high-resolution ADC is required. Typically, the ADC resolution for mobile health devices is 10–12 bits, however, a 12-bit ADC resolution was required to ensure that the developed system was future-proof. In addition to an on-board ADC, the microcontroller must have at least 8 digital IO pins, with at least three of these enabling pulse width modulation (PWM) to enable the system to work with a wide range of input/output devices. Bluetooth and WiFi capabilities were other requirements for the chosen microcontroller to enable easy communication between the designed system and the peripheral devices. Table 3 lists the specifications of each of the Arduino platforms that were evaluated. In the literature, while Arduino Uno [38,42], and Arduino Mega [43,44,45] are the most popular microcontroller options for usage with the AD8232, we have discounted Mega owing to its large footprint. We have implemented the prototype on an Arduino MKR 1010 (Arduino, Italy) owing to its 12-bit resolution, large flash memory, in-built Bluetooth feature, multiple PWM-enabled pins (see Table 3) and the fact that it is about ten years newer than other models, and to the best of our knowledge, has not been evaluated for ECG signal processing.

### 2.3. Additional Components

#### 2.3.1. Temperature Sensor

An infrared temperature sensor is necessary for non-contact temperature measurements. We have utilised MLX90614, a medical-grade Arduino-compatible sensor with a 0.02 °C resolution [46] and an accuracy of ±0.2 °C (Melexis, Belgium). It is a small, low-cost device with power-saving modes, operates via the I2C protocol, and has been used in earlier works as well [47,48].

#### 2.3.2. Blood Oxygen Sensor

In view of the COVID-19 pandemic, the measurement of the specific blood oxygen level (SpO_2_) is extremely important, and was included in the overall device to produce a multi-functional product. From the literature review of such SpO_2_ sensors, a range of products from Maxim Integrated were found to be available (a comparison can be seen below in Table 4). We have utilised the MAX30100 sensor, which has a small breakout board [49], and as compared to MAX30102, has a smaller footprint (Maxim Integrated, San Jose, CA, USA). The sensor is cost-effective, with a low-power mode for reduced current consumption during the sleep mode.

### 2.4. Data Output and Communications

As stated in the previous section, the system should provide a live ECG signal on a local display. For such displays, the Organic Light Emitting Diodes (OLED) and Liquid Crystal Display (LCD) are the two most common options for Arduino. While the OLED displays are emissive, which means they generate their light, the LCDs are non-emissive and require a backlight [50]. If only the heart rate were to be displayed to the user, the LCD display would have been optimal, however, as the live ECG signal is to be displayed, the 1.3” OLED with the highest resolution (see Table 5) and lowest power consumption is considered to be ideal [10].

#### 2.4.1. Bluetooth Mobile Application

The live ECG signal should also be presented on a mobile application, with the data being transmitted through Bluetooth in addition to the OLED display. The Arduino MKR 1010 has a built-in Bluetooth Low Energy (BLE) feature, making it an excellent choice for this work. However, it should be noted that mobile health services and applications should be driven by the use of the lowest common denominator technology, such as classic Bluetooth, which is supported by a higher percentage of mobile phones (as compared to BLE), particularly in LMICs [51]. Given that Android phones hold around 73% of the global mobile operating system market as of June 2021 [52], particularly in LMICs, a no-code approach was utilised to design an application compatible with Android phones. For this, the MIT App Inventor, a web-based, open-source development tool that offers a graphical interface for creating fully working Android apps, was chosen [53]. The finished software will have a primary screen that will show the user the live ECG signal as well as the patient’s heart rate, blood oxygen level, and body temperature. It will work with any smartphone that has Android 2.3 Gingerbread or a higher version.

#### 2.4.2. Web Server

A web server was added with the Bluetooth programme to monitor information such as heart rate, SpO_2_ level, and body temperature. Arduino IoT [54], Thingworx [55], and ThingSpeak [56] are examples of pre-made systems that offer such functionality. Since ThingSpeak can execute the MATLAB code, allowing for live signal processing and analysis, it was therefore chosen as the IoT server for patient data monitoring. It also makes it simple to import the sensor data into MATLAB. The Arduino MKR 1010 has a u-blox NINA-W102 multi radio module multi-radio that also supports WiFi connectivity, eliminating the need for a separate WiFi shield. The capability of reading raw ECG data from the AD8232 into MATLAB (through USB Serial) through the Arduino MKR 1010 has been introduced to the system.

## 3. System Overview

### 3.1. Component Block Diagram

**Figure 2:**
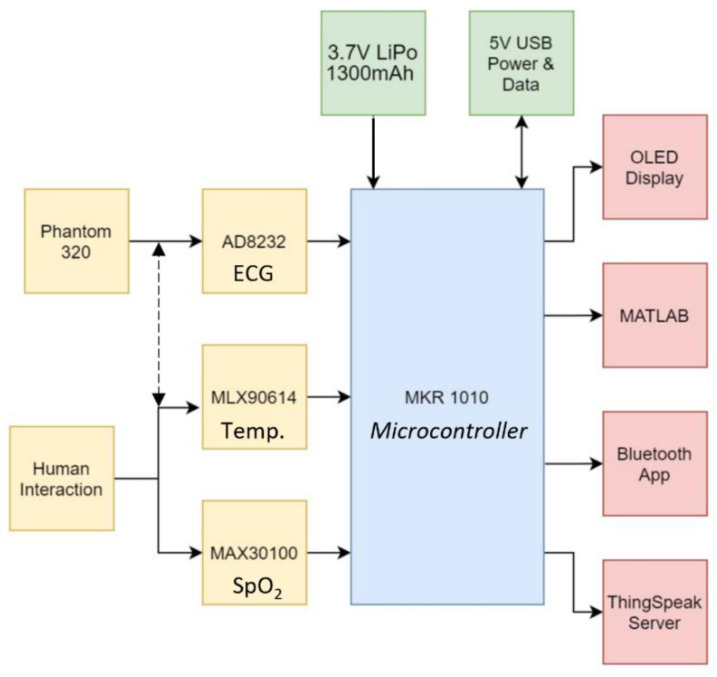
Component block diagram. The dotted line represents that ECG can be acquired either from a simulated source or from a human.

The inputs and outputs, as well as how they are interfaced with the microcontroller, are shown in Figure 1, and they are further described in the block diagram below (Figure 2). The MKR 1010 is powered by a USB 5 V or a 3.7 V LiPo battery, and the Phantom 320 (Medtec Science GMBH, Germany) generates the raw ECG signal, which is then processed by the AD8232 before entering an ADC pin on the MKR 1010. It should be noted that the ECG signal can also be captured from a live subject (as denoted by the dashed line). Their blood oxygen levels, and skin temperature may be measured and sent to MKR 1010 via contact with the human. A local OLED, a Bluetooth application, and MATLAB are used to display the processed ECG signal, while the basic patient data are shown using the ThingSpeak server.

### 3.2. System Flow Design

**Figure 3:**
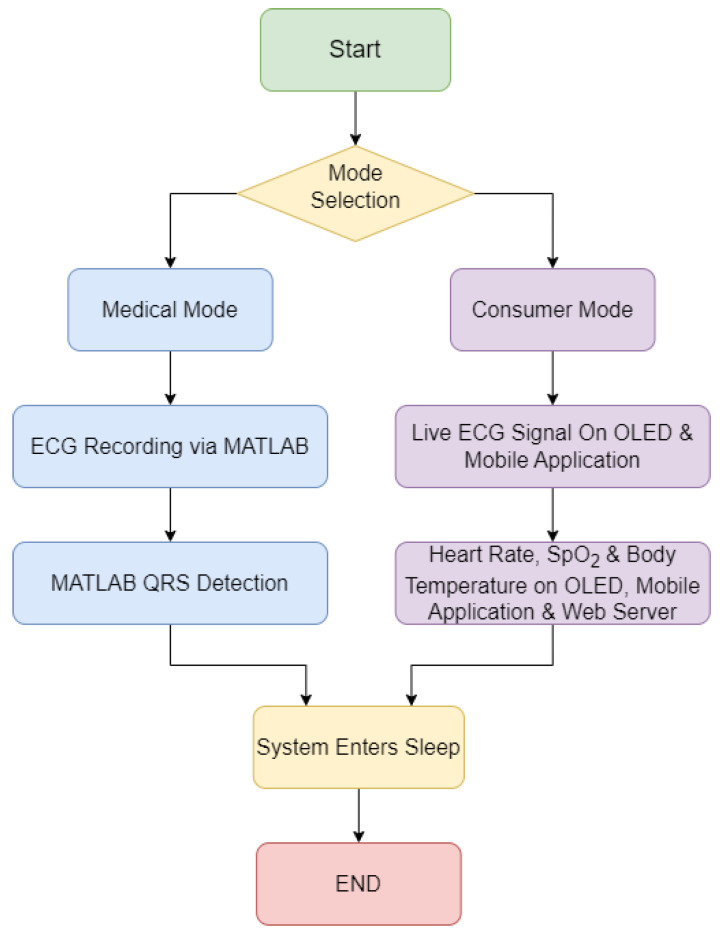
System flow diagram.

Following the selection of the hardware, the following phase in the design process was to determine how the system should function. The fundamental system flow diagram for the two developed modes, the consumer, and medical modes, is shown in Figure 3. The medical mode was created with MATLAB in mind for an extensive heart condition analysis. The consumer mode, on the other hand, is designed for the regular user who wants to track their heart rate, SpO_2_ levels, and body temperature and examine the information on an OLED display, a mobile phone app, or a web server.

## 4. PCB Design

**Figure 4:**
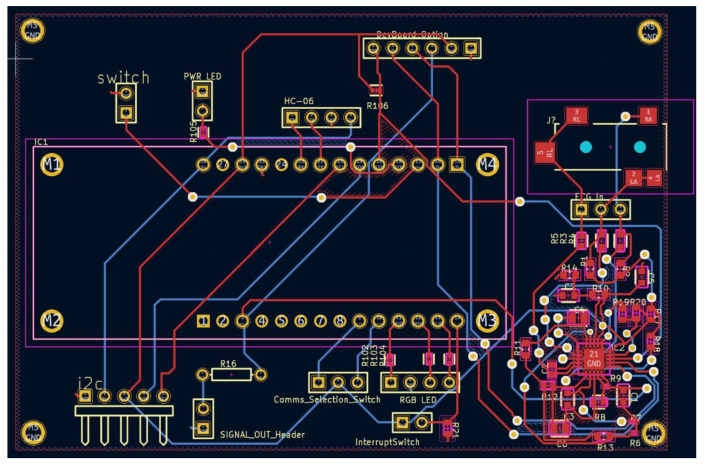
PCB layout highlight the routing and placement of various components.

Once the hardware and system design were completed, the next stage was to construct a breadboard-based circuit and to follow it up with the corresponding PCB design. The prototype PCB with the dimensions of 140 × 100 × 0.8 mm (Figure 4) had an MKR 1010 positioned in the center of the PCB to make track routing for extra components easier. It was linked through two 14-pin female headers onto which the MKR 1010 was inserted. The I2C line was used to link the MLX90614, MAX30100, and OLED. For the I2C line and 3.3 V, the MLX90614 (Melexis, Belgium) required two 10 KΩ pull-up 0402 resistors. The I2C lines and 3.3 V were necessary for the MAX30100 breakout board to function. For it to work, the OLED required I2C lines and 5 V. For these connections, female headers were installed on the PCB. A 4-pin female header was used to link the Bluetooth classic module to the MKR 1010 via SPI and 5 V line.

Two female headers for the switches were included on the PCB. A 3-pin header with two digital pins and 3.3 V was required for the mode selection switch. A 10-KΩ 0402 resistor linked to the ground and a 3.3 V supply powered the interrupt switch, which was a 2-pin header attached to a digital pin. To limit the potential of noise interfering with the raw ECG input signal, the AD8232 was positioned next to the five-pole 3.5 mm surface-mounted audio jack in conjunction with the passive 0402 and 0603 components. The short tracks near the AD8232 were 0.25 mm wide, whereas the longer tracks were made to be 0.75 mm wide to ensure that they were not damaged during handling.

A two-pole 0.5 Hz high-pass filter was followed by a two-pole 40 Hz low-pass filter in the AD8232 circuit. The gain of the op-amp was set to 11, resulting in a total system gain of 1100. The following equation was used to create the high-pass filter:

(1) $$f_{c}=\frac{10}{2\pi \;\sqrt{\left(R1\right)\left(C1\right)\left(R2\right)\left(C2\right)}}$$

(2) $$f_{c}=\frac{10}{2\pi \;\sqrt{\left(10\times 10^{6}\right)\left(0.33\times 10^{-6}\right)\left(10\times 10^{6}\right)\left(0.33\times 10^{-6}\right)}}\;\mathrm{where}\;f_{c}=0.48\;\mathrm{Hz}$$

The low-pass filter was designed using the following equation:

(3) $$f_{c}=\frac{1}{2\pi \;\sqrt{\left(R1\right)\left(C1\right)\left(R2\right)\left(C2\right)}}$$

(4) $$f_{c}=\frac{1}{2\pi \;\sqrt{\left(1\times 10^{6}\right)\left(10\times 10^{-9}\right)\left(1\times 10^{6}\right)\left(1.5\times 10^{-9}\right)}}\;\mathrm{where}\;f_{c}=41.09\;\mathrm{Hz}$$

(5) $$Gain_{LP}=1+\frac{R3}{R4}$$

(6) $$Gain_{LP}=1+\frac{1\times 10^{6}}{100\times 10^{3}}=11$$

As such, the complete electronic bill of materials (BOM) is shown in Table 6.

## 5. Arduino Code Design

### 5.1. System Flow Design

**Figure 5:**
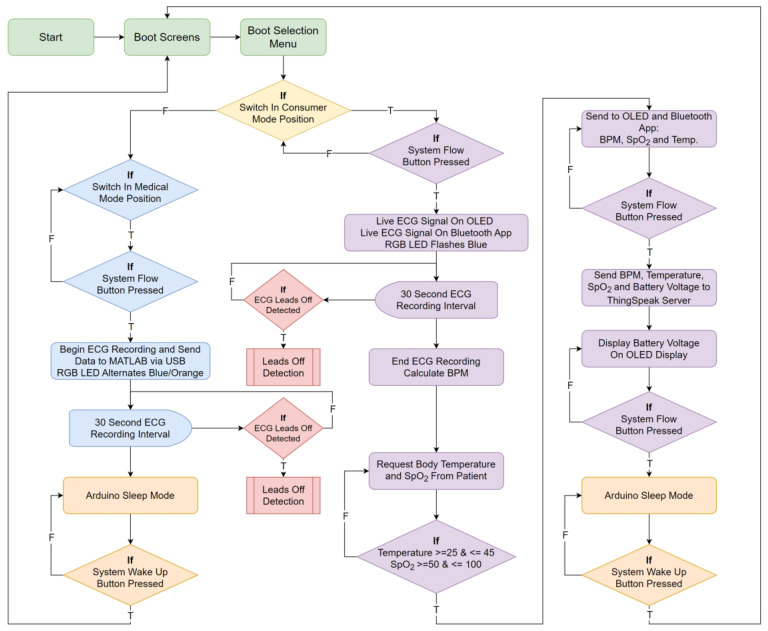
System level flow diagram. “F” represents the action if the condition is false, while “T” represents the action if the statement is true.

The final system flow diagram was developed prior to building the software for the MKR 1010, providing a full overview for completing the code. Figure 5 shows the flow diagram for the system operation. The two major modes, medical and consumer, both record the ECG signal for 30 s. When the device is in medical mode, the user is provided with graphs and charts, highlighting the identified QRS complexes, and when the ECG recording is completed, and the Arduino system goes to sleep. The live ECG signal is shown on both the OLED and Android apps when it is recording in the consumer mode. The patient’s SpO_2_ level and body temperature, as well as the computed heart rate and the voltage level of the on-board LiPo battery, are recorded and relayed to the OLED display, Android application, and the ThingSpeak Web Server, simultaneously, when the ECG recording is completed. The system will then go into sleep mode and stay there until the push button interrupt is used to wake it up. This is true in both the medical and consumer settings.

### 5.2. Code Design

The following sections provide a brief introduction of some of the codes’ most important features.

#### 5.2.1. OLED Live ECG Signal

One of the main features of the system is that it displays the live ECG signal on the OLED display during the consumer mode. When the analogRead() command is used to import the ECG signal from the AD8232 (10-bit ADC for consumer mode), the map function is used to reformat the data so that they may be shown on the OLED. The ‘drawLine’ command from the ‘u8g2’ library is then used to construct the ECG plot. The current and prior ‘*x*’ and ‘*y*’ values are among the four variables. This provides two co-ordinates between which a line can be drawn. When the value of ‘*x*’ surpasses 127 (the OLED is 128 pixels wide), the OLED is cleared, and graphing resumes on the left side of the display, moving towards the right.

#### 5.2.2. Lead-Off Detection

**Figure 6:**
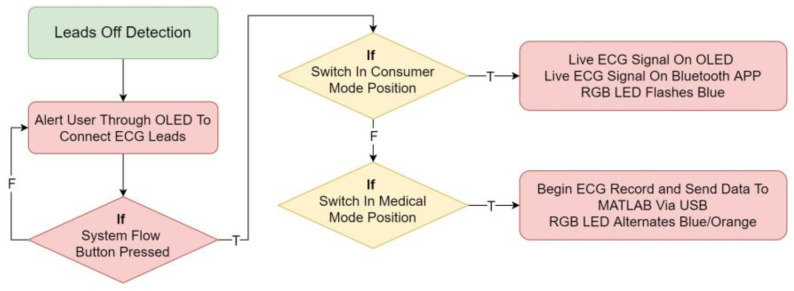
Lead off detection subroutine. “F” represents the action if the condition is false, while “T” represents the action if the statement is true.

It is critical to verify that there is no distortion between the body and the electrode while it is measuring a patient’s ECG signal, otherwise the stated findings may not correctly reflect the patient’s genuine cardiac status due to distortion. Techniques such as lead-off detection are recommended for ensuring that the electrodes are correctly attached to the patient, and it will alert the user/medical worker if they are not. The lead off detection subroutine as implemented on Arduino is shown in Figure 6.

#### 5.2.3. Sleep Mode

The addition of a sleep mode is beneficial in extending the battery source’s longevity as the system is meant for portable use. The ‘ArduinoLowPower’ library, which is meant for use with SAMD compatible boards such as the MKR1010 one, has been used to construct a hardware interrupt. At the end of both the medical and consumer modes, the machine goes into sleep mode. The function ‘wakeUpArduino’ executes whenever the low power interrupt is hit, and the system reboots via an internal reset.

#### 5.2.4. MATLAB Data Import

The ECG signal must be imported into MATLAB to implement the medical mode as previously described. The ‘Serial.print()’ method in Arduino is used to do this. In this case, it is utilised to communicate the ECG signal over USB to a PC running MATLAB. A 12-bit ADC resolution is employed in the medical mode to provide a higher signal accuracy. MATLAB additionally requires ‘Carriage Return’ and ‘Linefeed’ terminators.

## 6. Android Application

**Figure 7:**
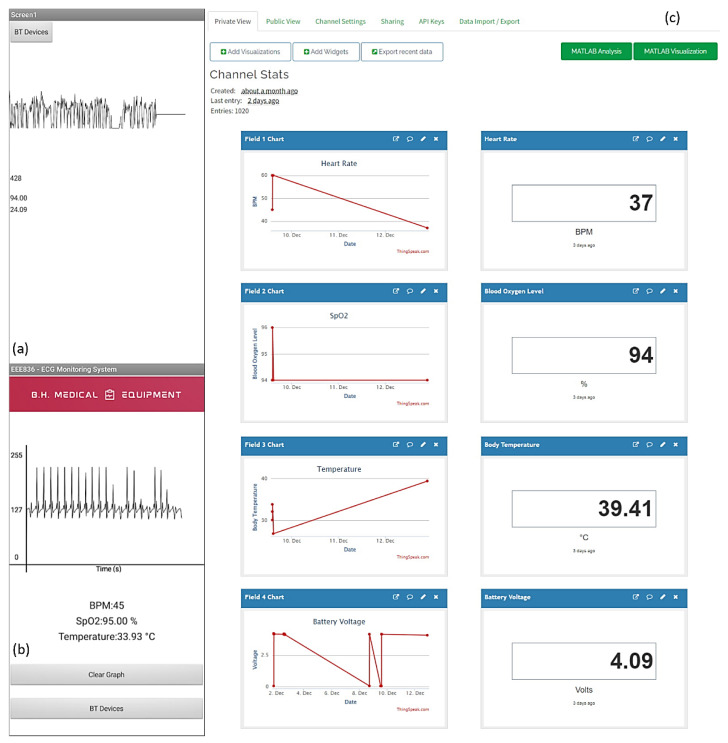
(**a**) Android App V1.0, (**b**) Android App V2.4, and (**c**) the ThingSpeak channel.

The Android application contains a real-time graphing of the recorded ECG signal, as well as displays of the heart rate, blood oxygen level, and body temperature. Figure 7a displays an early version of the Android app with a noisy ECG signal and minimal human–machine interaction elements. This was enhanced until the final application, Figure 7b, incorporated real-time ECG graphing and additional health care statistics.

### ThingSpeak

A ThingSpeak server, as shown in Figure 7c, was created as part of the work to allow for the remote monitoring of the patient’s heart rate, SpO_2_, and temperature levels, as well as the voltage of the internal LiPo battery. A channel was constructed in ThingSpeak to display these data, with each variable displayed on a graph and a text box displaying the last value received from the Arduino. The data were transferred wirelessly from the MKR 1010 to the ThingSpeak server via an API key for publishing to the server as well as the channel ID. The ‘write2TSData’ method, a part of the Arduino ThingSpeak library, was used to transmit the variables to the server.

## 7. ECG Signal Processing

As cardiovascular disease is the leading cause of mortality worldwide, substantial research and development into the diagnosis and prevention of CVD-related fatalities has been conducted [57]. The most important element of an ECG signal is the QRS complex. The QRS complex explains the electrical activity that occurs during Ventricular Depolarization, and it has been used to diagnose a variety of cardiac diseases. Throughout the 20th century, computer-based ECG equipment grew in popularity, and a survey in 1988 found that over half of the 100 million ECGs recorded in the United States were interpreted by computer ECG programs [58]. The advantages of computerised ECGs much outweighed the additional expense. The problem that hospitals faced was that there were just not enough electro cardiographers to read all of the ECGs that were being generated. The computerization of ECGs has substantially shortened the time between recording an ECG and diagnosing a patient [59].

Software development for QRS complex detection has been investigated for over 30 years, however, the computers’ capabilities limited any methods developed in the early phases. During the evolution of the microprocessor, the attention shifted away from hardware implementations and toward more software-based solutions for QRS detection [60]. For QRS detection, a variety of methods are utilised, including Pan-Tompkins, Hillbert Transform, and Wavelets Transform. A majority of these algorithms have a pre-processing step, which is followed by a decision stage that contains r-peak detection and decision logic. The focus these days is on AI-based systems that employ Convolutional Neural Networks (CNN) to recognise QRS complexes properly. The project ‘Common Standards for Quantitative Electrocardiology’ was a huge step forward in ECG signal processing. Many ECG datasets were created as part of this research, allowing for the extensive testing and development of new technologies. Some of the CSE project’s specifications are still relevant today [61].

### QRS Complex Detection Algorithm

A basic Pan–Tompkins algorithm was constructed in MATLAB to demonstrate the capabilities of the developed equipment [62]. The Pan–Tompkins algorithm relies on the detection of the QRS complex via the strongest observable feature which commences once the ECG signal is recorded into MATLAB using the ‘Medical Mode’. The following steps are outlined.

Filtering—To eliminate the typical components of noise present in ECG data, a bandpass filter of total order four with a passband of 5–15 Hz is used.

Differentiation—The filtered signal is then differentiated, yielding information about the QRS slopes.

Squaring—Entails squaring the differentiated signal. This increases the signal’s dominance of the frequencies known to include QRS information. It also lowers the chances of a T-wave being misidentified as an R-wave.

Integration—A moving mean integration window is used to integrate the squared signal. There are 54 samples in this window (150 ms). The QRS complexes will be distorted if the window is too large or too narrow, resulting in erroneous peak detections.

**Figure 8:**
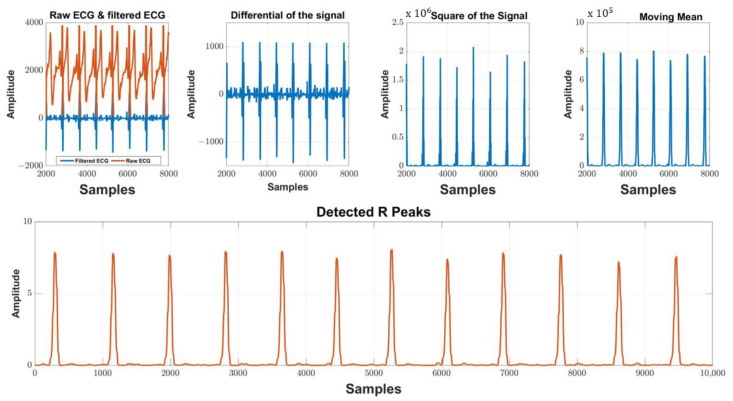
MATLAB ECG signal processing outputs. ×10^5^ and ×10^6^ are the scales of amplitude in the subplots.

Peak detection—As the simulator, Phantom 320, produces a clear signal, sophisticated thresholding requirements are unnecessary. A simple ‘findpeaks’ function may be used to detect the R waves in the ECG data at that time. If the gadget is to be commercialised, a more complex algorithm needs to be incorporated. Figure 8 illustrates the technique outlined above graphically.

**Figure 9:**
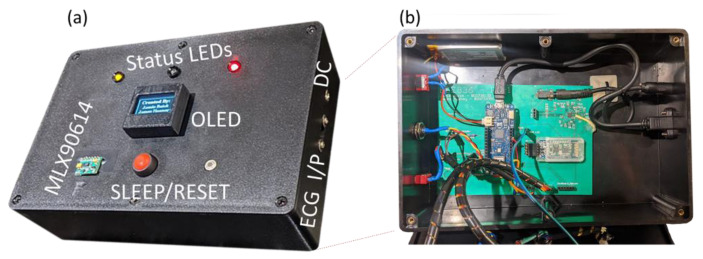
Final working prototype, (**a**) external packaging, and (**b**) internal structure.

Besides the ECG capture accuracy, a basic performance test was performed to evaluate the accuracy of the SpO_2_ sensor and the temperature sensor. The MAX30100 and the off-the-shelf Pulse Oximeter were used to collect a sample from the individual (Figure 9). The MAX30100 employed inside our developed system was 99% accurate in this testing situation, according to the results. Similarly, a performance test was performed to check the accuracy of the body temperature sensor. The MLX90614 and an infrared thermometer (Boots, United Kingdom) were used to record each patient’s body temperature. Table 7 summarises these findings.

## 8. Conclusions

In this study, we propose a pervasive healthcare service based on IoT-based ECG and vitals monitoring system. This system continually monitors the patients’ signs, such as ECG and SpO_2_, and offers data transmission modalities to balance the healthcare demand for communication and processing the resources. A prototype fulfilling the requirements of the amplification, filtering, digitization, processing, and transmission of the ECG signal was developed which further transmits it to multiple back-end devices including the displays, mobile applications, and web servers. In addition, the system keeps track of the SpO_2_ levels and body temperature. It generates precise ECG signal recordings while remaining portable as well as user-friendly. Invariably, in its current state-of-the-art form, the wearables technology (including smartwatches, smart vest, etc.) for ECG are more expensive than the portable ECG monitors are, and is therefore, they are not being considered for use in the context of LMICs, and thus, our system could provide a solution. In its current format, our proposed solution is similar to the other devices (shown in Table 1) which are capable of recoding 25–30 s of ECG data, and they often provide data on heart rate (variability) with the additional features of a smartphone interface and/or display screen and cloud integration. The cheapest option with all-inclusive features is ~$80, however, at such a price point, the end user’s affordability often becomes the constraint for their use in LMICs. We believe that scaling up further design iterations will bring down our cost down dramatically, and the further integration of Data Stream Management System (DSMS) technology will enhance capabilities such as continuous query, windowing, and aggregation. Following that, data stream mining and context awareness technologies are being evaluated as ways to give more powerful ubiquitous healthcare services to patients, such as early warning and real-time knowledge assistance.

## Figures and Tables

**Table 1:** Comparison of commercially available ECG capturing systems (√ means that the feature is available, while ✗ means that the feature is unavailable).

ECG Device	Single-Lead	Display Capability	Storage Facility	Built-in Battery	Data Transmission	Cloud-Based Services	Smart-Phone Interface	FDA Approval	Cost ($US)
Kardia mobile	√	√	✗	√	√	✗	√	√	$89
Omron HS	√	√	√	✗	√	✗	✗	✗	$387
Heal Force	√	√	√	√	√	√	✗	√	$170
Instant Check	√	√	√	√	✗	✗	✗	√	$422
Heart Check CardiBeat	√	✗	√	√	√	√	√	√	$129
Nuvant mobile	√	✗	√	√	√	√	√	√	$754
Afibalert	√	√	✗	✗	✗	✗	✗	√	$449
ECG Check	√	√	√	√	√	√	√	√	$80
Dimetek	√	√	√	√	✗	✗	✗	✗	NA
Ziopatch	√	✗	√	√	√	√	√	√	NA
Zenicore	√	✗	√	√	√	√	✗	✗	NA
Reka E100	√	✗	√	√	√	√	√	✗	NA
Read my heart	√	√	√	√	√	✗	✗	√	NA
Medtronic Reveal	√	✗	√	√	√	√	✗	✗	NA
Smart-vest (multi-lead)	✗	✗	✗	√	√	√	√	✗	NA
This work	√	√	√	√	√	√	√	✗	$131

**Table 2:** Analog front-end IC comparison (√ means that the feature is available).

Parameter	AD8232 [39]	HM301D [40]	ADS1191 [41]
Manufacturer	Analog Devices	ST Microelectronics	Texas Instruments
Dimensions (mm)	4 × 4	6 × 6	5 × 5
Operating Voltage	2–3.5 V	1.62–3.6 V	1.7–3.6 V
Operating Current (operating power)	170 µA	1.3 mA	(335 µW/channel)
Output Impedance	10 GΩ	50 MΩ	100 MΩ
Gain	100 V/V	64 V/V	12 V/V
Low Power Mode	√	√	√
Leads-off Detection	√	√	√
ECG Channel	1	3	2
Chip Cost	$5.00	$5.00	$5.00

**Table 3:** Arduino hardware options (√ means that the feature is available, while ✗ means that the feature is unavailable).

Parameter	Arduino Uno	Arduino Nano	Arduino MKR 1010
Dimensions (mm)	68.6 × 53.4	45 × 18	61.5 × 25
Processor	ATmega328P	ATmega328	SAMD21
Clock Speed	16 MHz	16 MHz	48 MHz
Flash Memory	32 KB	32 KB	256 KB
SRAM	2 KB	2 KB	32 KB
Digital I/O Pins	14	22	21
Digital PWM Pins	6	6	13
Analog Input Pins	6	8	7
ADC Resolution	10-bit	10-bit	12-bit
WiFi	✗	✗	√
Bluetooth	✗	✗	√
On-Board Charging	✗	✗	√
Price	£20.00	£18.00	£27.90
Release Date	September 2010	May 2008	June 2018

**Table 4:** SpO_2_ and temperature sensor options (√ means that the feature is available, while ✗ means that the feature is unavailable).

-	SpO_2_ Sensor			Temperature Sensor		
Parameter	MAX30100	MAX30101	MAX30102	DS18B20	MLX90614	DHT11
Manufacturer	Maxim Int.	Maxim Int.	Maxim Int.	Maxim Int.	Melexis	
Dimensions (mm)	5.6 × 2.8 × 1.2	5.6 × 3.3 × 1.5	5.6 × 3.3 × 1.5		17.10 × 10 dia.	15.5 × 12 × 5.5
Operating Voltage	3.3 V	5 V	3.3 V	3–5.5 V	3.3 V	3.5–5.5 V
Operating Current	600 µA	600 µA	600 µA	1 mA	1.5 mA	300 µA
Low Power Mode	√	√	√		✗	√
Low Power Current	0.7 µA	0.7 µA	0.7 µA		-	60 µA
Interface	I2C	I2C	I2C	1-wire	I2C	I2C

**Table 5:** Display hardware options.

**Parameter**	16 × 2 LCD	1.3” OLED	0.91” OLED
**Resolution**	16 × 2	128 × 64	128 × 32
**Interface**	I2C	I2C	I2C
**Power Consumption**	50 mA	11 mA	20 mA
**Operating Temperature**	−10 to 60 °C	−20 to 70 °C	−40 to 85 °C

**Table 6:** Electronic components BOM.

Component	Description	Designator	Quantity	Unit Price (£)	Total Price (£)
GRM1885C1H152JA01D	CAP 0603, 1500 pF, 50 V	C1	1	0.045	0.045
1 nF	CAP 1 nF 50 V 0603	C2	1	0.38	0.38
CC0603KRX7R9BB103	CAP 10000PF 50 V 0603	C3	1	0.82	0.82
C0805C334K4RACTU	CAP 0.33 UF 16 V 0805	C4, C6	2	0.0912	0.018
CC0603KRX7R7BB104	CAP 0.1 UF 16 V 0603	C5, C7	2	0.053	0.106
3-Pin Switch Mode type	Header, 3-Pin	Mode selection pin	1	0.58	0.58
ECG in header	Header, 3-Pin	ECG In	1	1.72	1.72
AD8232 Dev Board option	Header, 6-Pin	DevBoard Option	1	0.64	0.64
BT module	Header, 4-Pin	HC-05	1	6.99	6.99
MKR	MKR1010	IC1	1	25.00	25.00
AD8232ACPZ-R7	Integrated Circuit	IC2	1	4.85	4.85
Oxygen Sensor	Header, 7-Pin	IC3	1	4.45	4.45
Interrupt Switch	Header, 2-Pin	Interrupt switch	1	0.58	0.58
Audio Jack for ECG in	35RASMT4BHNTRX	J1	1	3.00	3.00
LCD Display	Header, 4-Pin	LCD	1	6.49	6.49
Signal out LED	Header, 2-Pin	Signal Out Header	1	0.24	0.24
Power LED	Header, 2-Pin	PWR LED	1	0.24	0.24
1RT0603BRD07180KL	RES 180 K 0603	R3, R4	2	0.10	0.20
CRCW0603360KFKEA	RES 360K 0603	R5	1	0.10	0.10
1 M	RES 1 M 0201	R6, R7, R9	3	0.10	0.30
CRCW0603100KFKEA	RES 100 k 0603	R8	1	0.097	0.097
1.4 M	RES 1.4 M 0603	R12	1	0.086	0.086
DNF	DNF	R16	1	-	-
RC0603JR-070RL	RES, 0, 0603	R17, R19	2	0.086	0.172
DNF	DNF	R18, R20	2	-	-
RC0603FR-0710KL	RES, 10 k, 0603	R21	1	0.10	0.10
CRCW040210K0FKED	RES 10 K 0402	R100, R101	2	0.10	0.20
CRCW0402220RFKED	RES 220, 0402	R102, R103, R104, R105	4	0.10	0.40
LED2	Header, 4-Pin	RGB LED	1	0.99	0.99
MLX90614	Infrared temperature sensor	Temp	1	30.31	30.31
1300 mAh LiPo	-	Batt	1	17.39	17.39
Micro USB Extension	-	USB EXT	1	3.49	3.49
Audio Jack Extension	-	Audio EXT	1	2.68	2.68

**Table 7:** Body temperature and SpO_2_ sensor performance testing performance testing.

Subject	Infrared Thermometer	MLX90614	Pulse Oximeter	MAX30100
Patient 1 Patient 2 Patient 3	32.0 °C 34.5 °C 33.8 °C	33.4 °C 35.8 °C 34.7 °C	96% 94% 94%	94% 96% 94%

## References

[1] Swaroop K.N., Chandu K., Gorrepotu R., Deb S. (2019). A Health Monitoring System for Vital Signs Using IoT. Internet Things.

[2] Thwaites C.L., Ngoc Dinh M., Nygate J., Hoang Minh Tu V., van Cuong N., Anh T.T., McBride A., Huynh T., Chau N.H., Lâm H.M. (2020). New Technologies to Improve Healthcare in Low- and Middle-Income Countries: Global Grand Challenges Satellite Event, Oxford University Clinical Research Unit, Ho Chi Minh City, 17th-18th September 2019. Wellcome Open Res..

[3] Soin N., Fishlock S.J., Kelsey C., Smith S. (2021). Triboelectric Effect Enabled Self-Powered, Point-of-Care Diagnostics: Opportunities for Developing ASSURED and REASSURED Devices. Micromachines.

[4] Cardiovascular Diseases. https://www.who.int/health-topics/cardiovascular-diseases#tab=tab_1.

[5] Roth G.A., Johnson C.O., Abate K.H., Abd-Allah F., Ahmed M., Alam K., Alam T., Alvis-Guzman N., Ansari H., Ärnlöv J. (2018). The Burden of Cardiovascular Diseases Among US States, 1990-2016. JAMA Cardiol..

[6] Mozaffarian D., Benjamin E.J., Go A.S., Arnett D.K., Blaha M.J., Cushman M., de Ferranti S., Després J.P., Fullerton H.J., Howard V.J. (2015). Executive Summary: Heart Disease and Stroke Statistics-2015 Update: A Report from the American Heart Association. Circulation.

[7] Nichols M., Townsend N., Scarborough P., Rayner M. (2014). Cardiovascular Disease in Europe 2014: Epidemiological Update. Eur. Heart J..

[8] Anand S., Bradshaw C., Prabhakaran D. (2020). Prevention and Management of CVD in LMICs: Why Do Ethnicity, Culture, and Context Matter?. BMC Med..

[9] Navarro C., Fishlock S.J., Steele D.N., Puttaswamy S.V., Lubarsky G., Raj S., McLaughlin J. (2020). A Point-of-Care Measurement of NT-ProBNP for Heart Failure Patients. IEEE Access.

[10] Faruk N., Abdulkarim A., Emmanuel I., Folawiyo Y.Y., Adewole K.S., Mojeed H.A., Oloyede A.A., Olawoyin L.A., Sikiru I.A., Nehemiah M. (2021). A Comprehensive Survey on Low-Cost ECG Acquisition Systems: Advances on Design Specifications, Challenges and Future Direction. Biocybern. Biomed. Eng..

[11] Chamadiya B., Mankodiya K., Wagner M., Hofmann U.G. (2013). Textile-Based, Contactless ECG Monitoring for Non-ICU Clinical Settings. J. Ambient. Intell. Humaniz. Comput..

[12] Kwon J.M., Jeon K.H., Kim H.M., Kim M.J., Lim S.M., Kim K.H., Song P.S., Park J., Choi R.K., Oh B.H. (2020). Comparing the Performance of Artificial Intelligence and Conventional Diagnosis Criteria for Detecting Left Ventricular Hypertrophy Using Electrocardiography. EP Eur..

[13] Siva Nagendra Reddy P., Vishnu Vardhan D., Tharun Kumar Reddy K., Ajay Kumar Reddy P. (2018). An IoT-Based Low-Cost Weather Monitoring and Alert System Using Node MCU. Smart Innov. Syst. Technol..

[14] Hutchison A.W., Malaiapan Y., Jarvie I., Barger B., Watkins E., Braitberg G., Kambourakis T., Cameron J.D., Meredith I.T. (2009). Prehospital 12-Lead ECG to Triage ST-Elevation Myocardial Infarction and Emergency Department Activation of the Infarct Team Significantly Improves Door-to-Balloon Times: Ambulance Victoria and Monash Heart Acute Myocardial Infarction (MonAMI) 12-Lead ECG Project. Circ. Cardiovasc. Interv..

[15] Fensli R., Gunnarson E., Gundersen T. A Wearable ECG-Recording System for Continuous Arrhythmia Monitoring in a Wireless Tele-Home-Care Situation. Proceedings of the 18th IEEE Symposium on Computer-Based Medical Systems.

[16] Gionfriddo W.J., Laidlaw D.W., Mark Estes N.A. (2022). Ambulatory Electrocardiography. Cardiology Procedures.

[17] Petrenas A., Marozas V., Jaruševičius G., Sörnmo L. (2015). A Modified Lewis ECG Lead System for Ambulatory Monitoring of Atrial Arrhythmias. J. Electrocardiol..

[18] Elgendi M., Al-Ali A., Mohamed A., Ward R. (2018). Improving Remote Health Monitoring: A Low-Complexity ECG Compression Approach. Diagnostics.

[19] Guan K., Shao M., Wu S. (2017). A Remote Health Monitoring System for the Elderly Based on Smart Home Gateway. J. Healthc. Eng..

[20] Selvaraj S., Sundaravaradhan S. (2020). Challenges and Opportunities in IoT Healthcare Systems: A Systematic Review. SN Appl. Sci..

[21] Tuli S., Basumatary N., Gill S.S., Kahani M., Arya R.C., Wander G.S., Buyya R. (2020). HealthFog: An Ensemble Deep Learning Based Smart Healthcare System for Automatic Diagnosis of Heart Diseases in Integrated IoT and Fog Computing Environments. Future Gener. Comput. Syst..

[22] Oueida S., Kotb Y., Aloqaily M., Jararweh Y., Baker T. (2018). An Edge Computing Based Smart Healthcare Framework for Resource Management. Sensors.

[23] Sodhro A.H., Luo Z., Sangaiah A.K., Baik S.W. (2019). Mobile Edge Computing Based QoS Optimization in Medical Healthcare Applications. Int. J. Inf. Manag..

[24] El-Rashidy N., El-Sappagh S., Riazul Islam S.M., El-Bakry H.M., Abdelrazek S. (2021). Mobile Health in Remote Patient Monitoring for Chronic Diseases: Principles, Trends, and Challenges. Diagnostics.

[25] Mamdiwar S.D., Akshith R., Shakruwala Z., Chadha U., Srinivasan K., Chang C.Y. (2021). Recent Advances on IoT-Assisted Wearable Sensor Systems for Healthcare Monitoring. Biosensors.

[26] Serhani M.A., El Kassabi H.T., Ismail H., Navaz A.N. (2020). ECG Monitoring Systems: Review, Architecture, Processes, and Key Challenges. Sensors.

[27] Vuorinen T., Noponen K., Vehkaoja A., Onnia T., Laakso E., Leppänen S., Mansikkamäki K., Seppänen T., Mäntysalo M. (2019). Validation of Printed, Skin-Mounted Multilead Electrode for ECG Measurements. Adv. Mater. Technol..

[28] Lv W., Guo J. (2021). Real-Time ECG Signal Acquisition and Monitoring for Sports Competition Process Oriented to the Internet of Things. Measurement.

[29] Carney R.M., Freedland K.E., Steinmeyer B.C., Rubin E.H., Stein P.K., Rich M.W. (2016). Nighttime Heart Rate Predicts Response to Depression Treatment in Patients with Coronary Heart Disease. J. Affect. Disord..

[30] Valenza G., Nardelli M., Lanata A., Gentili C., Bertschy G., Kosel M., Scilingo E.P. (2016). Predicting Mood Changes in Bipolar Disorder Through Heartbeat Nonlinear Dynamics. IEEE J. Biomed. Health Inform..

[31] Babusiak B., Borik S., Smondrk M. (2020). Two-Electrode ECG for Ambulatory Monitoring with Minimal Hardware Complexity. Sensors.

[32] Pitman B.M., Chew S.-H., Wong C.X., Jaghoori A., Iwai S., Thomas G., Chew A., Sanders P., Lau D.H. (2021). Performance of a Mobile Single-Lead Electrocardiogram Technology for Atrial Fibrillation Screening in a Semirural African Population: Insights from “The Heart of Ethiopia: Focus on Atrial Fibrillation” (TEFF-AF) Study. JMIR Mhealth Uhealth.

[33] Soni A., Earon A., Handorf A., Fahey N., Talati K., Bostrom J., Chon K., Napolitano C., Chin M., Sullivan J. (2016). High Burden of Unrecognized Atrial Fibrillation in Rural India: An Innovative Community-Based Cross-Sectional Screening Program. JMIR Public Health Surveill..

[34] Singh B.N., Tiwari A.K. (2006). Optimal Selection of Wavelet Basis Function Applied to ECG Signal Denoising. Digit. Signal Processing.

[35] Raja K., Saravanan S., Anitha R., Priya S.S., Subhashini R. Design of a Low Power ECG Signal Processor for Wearable Health System-Review and Implementation Issues. Proceedings of the 2017 11th International Conference on Intelligent Systems and Control, ISCO 2017.

[36] Gifari M.W., Zakaria H., Mengko R. Design of ECG Homecare:12-Lead ECG Acquisition Using Single Channel ECG Device Developed on AD8232 Analog Front End. Proceedings of the 5th International Conference on Electrical Engineering and Informatics: Bridging the Knowledge between Academic, Industry, and Community, ICEEI 2015.

[37] Agung M.A. (2017). Basari 3-Lead Acquisition Using Single Channel ECG Device Developed on AD8232 Analog Front End for Wireless ECG Application. AIP Conf. Proc..

[38] Iskandar W.J., Roihan I., Koestoer R.A. (2019). Prototype Low-Cost Portable Electrocardiogram (ECG) Based on Arduino-Uno with Bluetooth Feature. AIP Conf. Proc..

[39] Single-Lead, Heart Rate Monitor Front End. www.analog.com.

[40] HM301D-Diagnostic Quality Acquisition System for Bio-Electric Sensors and Bio-Impedance Measurements-STMicroelectronics. https://www.st.com/en/data-converters/hm301d.html.

[41] ADS1191 Data Sheet, Product Information and Support|TI.Com. https://www.ti.com/product/ADS1191.

[42] Dioren Rumpa L., Suluh S., Hendrika Ramopoly I., Jefriyanto W. (2020). Development of ECG Sensor Using Arduino Uno and E-Health Sensor Platform: Mood Detection from Heartbeat. J. Phys. Conf. Ser..

[43] Güvenç H. Wireless ECG Device with Arduino. Proceedings of the TIPTEKNO 2020-Tip Teknolojileri Kongresi-2020 Medical Technologies Congress, TIPTEKNO 2020.

[44] Bravo-Zanoguera M., Cuevas-González D., Reyna M.A., García-Vázquez J.P., Avitia R.L. (2020). Fabricating a Portable ECG Device Using AD823X Analog Front-End Microchips and Open-Source Development Validation. Sensors.

[45] Tătaru A.I. (2019). Drugă; CN Designing and Realization an ECG Based the Arduino Mega 2560 Development Board. IOP Conf. Ser. Mater. Sci. Eng..

[46] Digital Non-Contact Infrared Thermometer (MLX90614) #Melexis. https://www.melexis.com/en/product/mlx90614/digital-plug-play-infrared-thermometer-to-can.

[47] Marques G., Pitarma R. (2019). Non-Contact Infrared Temperature Acquisition System Based on Internet of Things for Laboratory Activities Monitoring. Procedia Comput. Sci..

[48] Jin G., Zhang X., Fan W., Liu Y., He P. (2015). Design of Non-Contact Infra-Red Thermometer Based on the Sensor of MLX90614. Open Autom. Control. Syst. J..

[49] MAX30100 Pulse Oximeter and Heart-Rate Sensor IC for Wearable Health|Maxim Integrated. https://www.maximintegrated.com/en/products/sensors/MAX30100.html.

[50] Optics & Photonics News-OLED Versus LCD: Who Wins?. https://www.optica-opn.org/home/articles/volume_26/february_2015/departments/oled_versus_lcd_who_wins/.

[51] van Heerden A., Tomlinson M., Swartz L. (2012). Point of Care in Your Pocket: A Research Agenda for the Field of m-Health. Bull. World Health Organ..

[52] Top A., Gökbulut M. (2022). Android Application Design with MIT App Inventor for Bluetooth Based Mobile Robot Control. Wirel. Pers. Commun..

[53] Mobile OS Market Share 2021|Statista. https://www.statista.com/statistics/272698/global-market-share-held-by-mobile-operating-systems-since-2009/.

[54] Singh P., Jasuja A. IoT Based Low-Cost Distant Patient ECG Monitoring System. Proceedings of the IEEE International Conference on Computing, Communication and Automation, ICCCA 2017.

[55] Kadarina T.M., Priambodo R. (2018). Monitoring Heart Rate and SpO2 Using Thingsboard IoT Platform for Mother and Child Preventive Healthcare. IOP Conf. Ser. Mater. Sci. Eng..

[56] Bharadwaj K., Dhawan R., Ray M.K., Mahalakshmi P. (2018). Wi-Fi-Based Low-Cost Monitoring of ECG and Temperature Parameters Using Arduino and ThingSpeak. Lect. Notes Electr. Eng..

[57] Liu F., Liu C., Jiang X., Zhang Z., Zhang Y., Li J., Wei S. (2018). Performance Analysis of Ten Common QRS Detectors on Different ECG Application Cases. J. Healthc. Eng..

[58] Drazen E., Mann N., Borun R., Laks M., Bersen A. (1988). Survey of Computer-Assisted Electrocardiography in the United States. J. Electrocardiol..

[59] Rautaharju P.M. (2016). Eyewitness to History: Landmarks in the Development of Computerized Electrocardiography. J. Electrocardiol..

[60] Köhler B.U., Hennig C., Orglmeister R. (2002). The Principles of Software QRS Detection. IEEE Eng. Med. Biol. Mag..

[61] Pan J., Tompkins W.J. (1985). A Real-Time QRS Detection Algorithm. IEEE Trans. Biomed. Eng..

[62] Rubel P., Fayn J., Macfarlane P.W., Pani D., Schlögl A., Värri A. (2021). The History and Challenges of SCP-ECG: The Standard Communication Protocol for Computer-Assisted Electrocardiography. Hearts.

